# Understanding nanoparticle flow with a new in vitro experimental and computational approach using hydrogel channels

**DOI:** 10.3762/bjnano.11.22

**Published:** 2020-02-06

**Authors:** Armel Boutchuen, Dell Zimmerman, Abdollah Arabshahi, John Melnyczuk, Soubantika Palchoudhury

**Affiliations:** 1 Department of Civil and Chemical Engineering, University of Tennessee at Chattanooga, Chattanooga, Tennessee 37403, United States; 2 SimCenter, University of Tennessee at Chattanooga, Chattanooga, Tennessee 37403, United States; 3 Department of Chemistry, Clark Atlanta University, Georgia 30314, United States

**Keywords:** computational fluid dynamics, drug delivery, iron oxide nanoparticles, nanoparticle flow, poly(hydroxyethyl methacrylate) (pHEMA) hydrogels

## Abstract

Nanoparticles (NPs) are considered as one of the most promising drug delivery vehicles and a next-generation solution for current medical challenges. In this context, variables related to flow of NPs such as the quantity of NPs lost during transport and flow trajectory greatly affect the clinical efficiency of NP drug delivery systems. Currently, there is little knowledge of the physical mechanisms dominating NP flow inside the human body due to the limitations of available experimental tools for mimicking complex physiological environments at the preclinical stage. Here, we report a coupled experimental and computational fluid dynamics (CFD)-based novel in vitro approach to predict the flow velocity and binding of NP drug delivery systems during transport through vasculature. Poly(hydroxyethyl)methacrylate hydrogels were used to form soft cylindrical constructs mimicking vascular sections as flow channels for synthesized iron oxide NPs in these first-of-its-kind transport experiments. Brownian dynamics and material of the flow channels played key roles in NP flow, based on the measurements of NP flow velocity over seven different mass concentrations. A fully developed laminar flow of the NPs under these conditions was simultaneously predicted using CFD. Results from the mass loss of NPs during flow indicated a diffusion-dominated flow at higher particle concentrations but a flow controlled by the surrounding fluid and Brownian dynamics at the lowest NP concentrations. The CFD model predicted a mass loss of 1.341% and 6.253% for the 4.12 g·mL^−1^ and 2.008 g·mL^−1^ inlet mass concentrations of the NPs, in close confirmation with the experimental results. This further highlights the reliability of our new in vitro technique in providing mechanistic insights of NP flow for potential preclinical stage applications.

## Introduction

Current research increasingly highlight the importance of drug delivery systems in engineering new solutions to our medical challenges [1–3]. A drug delivery agent should be biocompatible, easily administered to the patient, and capable of carrying the drug to the disease site before controlled release of the drug [4–5]. NPs, particularly magnetic iron oxide NPs, are highly attractive for drug delivery because they have a higher circulation time compared to the conventional drugs and can be easily delivered to the diseased location through passive, active, or physical targeting [6]. The NPs can essentially transport a large drug payload past the complex physiological microenvironment inside the human body to the target site. In cases of injection of the NPs to the blood stream, the particles must first flow through vascular regimes with high plasma protein concentrations, followed by transvascular transport through vascular networks of varying dimensions within the body, before reaching the action site [7–9]. It is increasingly complex to predict the flow properties of NP-based drug delivery system such as the local velocity and adhesion of the NPs in vivo. If we can predict the flow and interaction, e.g., adhesion or deposition, of the NPs through in vitro techniques, it will significantly enhance the preclinical to clinical translation of NP-based drugs from the current success rate of 8% [10]. Currently, preclinical assessment of NP drug delivery systems relies on animal models to provide a reliable mimic of conditions within the patient’s body [11]. Therefore, an in vitro technique that can mimic the transport of NPs through vascular constructs will be a major initial screening tool for a controlled patient-specific environment and will significantly complement existing in vivo methods for a more efficient and reliable evaluation of NP drugs [12]. However, many variables such as the size, surface chemistry, and interaction of the NPs with different biological compounds within the body influence the trajectory of NPs in vivo [13–15]. This makes it challenging to predict the behavior of NP drugs relying on solely experimental approaches [16].

To this end, computational methods have been used in modeling NP-based drug delivery through human vascular networks [17]. Different types of in silico methods have been investigated to date in an effort to cover the vastly different dimension scales of the NPs and the vascular network [18–20]. These simulations can essentially be categorized on the basis of details in the physics used to define and model the system [5]. Ab initio quantum mechanical simulations represent the highest level of detail, but are most applicable for modeling smaller NP systems or optimizing less detailed simulations due to their increased complexity and computational cost [17]. The coarse grained molecular dynamics simulations can characterize larger systems over time scales greater than 1 ms [21]. These slightly coarser models simulate a group of atoms or molecular fragment instead of an individual atom to incorporate a larger system. However, molecular dynamics models in general are more appropriate in understanding the size, surface, interaction, and uptake of a NP rather than their flow over larger dimensions as it models the interactions of molecules and atoms for a specific time scale. Beyond coarse grained models and molecular modeling, dissipative particle dynamics have been used to simulate the hydrodynamic properties of NPs over larger length scales [22–23]. However, even the dissipative particle dynamics models are limited in length scale in terms of simulating NP flow through vascular networks for drug delivery. Therefore, continuum models have been explored for NP transport [24]. For example, advection–diffusion models have been applied for transport of NPs through larger vascular constructs where the blood is represented as a simple Newtonian fluid [25–26]. The convection–diffusion-reaction continuum models were more suitable in terms of modeling the ligand–receptor binding reactions of NPs in transport through microscale vascular channels. Computational fluid dynamics (CFD), traditionally used in modeling fluid flow can be a powerful technique for continuum scale simulations of NP flow. The advantage of CFD in comparison to other computationally expensive techniques is the robustness and simplicity of the underlying physics [27–29]. CFD methods predict NP flow by solving the fundamental Navier–Stokes equation for flow [30–31]. Fullstone et al. have used CFD in couple with flexible large-scale agent based modeling to predict NP distributions in vivo [32]. The size and shape of the NPs greatly influenced the adhesion and path of NPs within the vascular network. This CFD model predicted a higher circulation time for the smaller NPs as these NPs had less chances of adhesion to the walls of the blood vessels, which was observed in some cases of in vitro experiments. The Brownian adhesion dynamics also plays an important role in NP binding compared to microscale structures [33]. Recently, we have developed CFD simulations to reliably model NP flow through plastic channels [34]. One key limitation of these few computational techniques used in understanding NP flow is the lack of suitable experimental data for validation. In order to acquire in vitro experimental results for transport of NPs that can reliably represent the flow and adhesion during drug delivery, it is essential to construct mimics of human vascular network.

Hydrogels are unique three-dimensional (3D) networks of insoluble polymers formed from two or more hydrophilic monomers that can absorb large quantities of water. The hydrogels, being generally soft, elastic, and biocompatible structures with a high water content are highly attractive from a materials perspective for making 3D channels mimicking the human vascular network [35–37]. Chemically cross-linked hydrogels such as poly(hydroxyethyl)methacrylate (pHEMA) are used as scaffold materials for the 3D bioprinting of biomimetic structures [38–39]. Therefore, we chose pHEMA hydrogels to build straight cylindrical channels mimicking sections of human vasculature for our experimental investigation of NP flow. In addition, we aimed to synthesize the hydrogel flow channels via a new, facile, and cost-effective approach.

Here, we report a combined experimental and computational technique to serve as an in vitro assessment of the flow of NPs using novel pHEMA hydrogel channels as the biomimetic flow path. The flow of NPs was experimentally investigated with different iron oxide NP formulations synthesized via a facile method for monitoring the velocity and mass loss of NPs through the hydrogel channels. The experimental flow of NPs was compared with computational predictions for a reliable assessment of NP flow through soft vascular mimics. An in silico CFD method using the Navier–Stokes-based Tenasi flow solver was applied to investigate the velocity and deposition of the NPs in our study. The results from the complementary experimental and CFD approach provided valuable understanding of the transport mechanisms dominating NP flow at different concentration regimes that has not been reported earlier.

## Results and Discussion

NPs such as biocompatible iron oxide NPs have been increasingly explored as carriers of different therapeutic agents to disease locations within the human body. NPs are attractive agents for such drug delivery applications due to their ability for enhanced circulation and controlled delivery to the disease sites compared to conventional drug formulations. In the context of achieving next-generation medical solutions with NPs, an in vitro technique that can mimic or visualize the transport of these NP-based drugs through vascular networks will be a key technological advancement. Such an in vitro method will serve as a complement for existing preclinical animal models and will be able to predict the flow of new NP drugs for increased success in clinical translation. A crucial first step for mimicking NP flow through human vascular network is developing the soft, 3D vascular structures. Hydrogels, soft biocompatible polymers with synthetically tunable physical and chemical properties and a chemical structure capable of retaining high volumes of water similar to the extracellular microenvironments, are considered highly promising materials for 3D vascular constructs. Therefore, we first synthesized a series of chemically cross-linked pHEMA hydrogels and investigated their structural properties and water absorption capability to find the best suitable formulation for fabricating flow channels mimicking a soft vascular platform. In this study, we aimed to design soft flow channels towards a more realistic mimic of biological systems such as vascular constructs for the experimental section of the analysis of NP flow.

**Figure 1:**
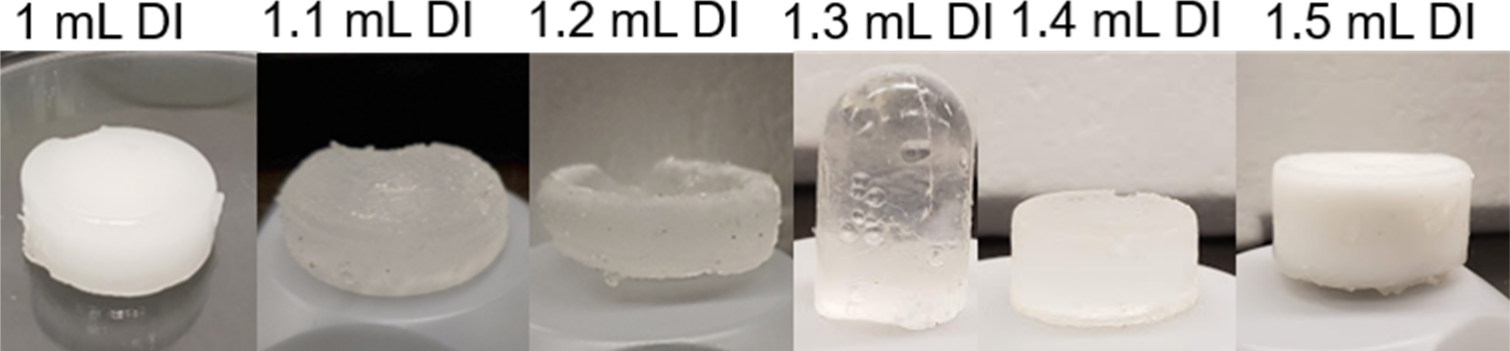
Images of pHEMA gels prepared with different quantities of DI water.

Figure 1 shows the images of freshly synthesized pHEMA gels prepared with different quantities of water (1–1.5 mL) using the same cross-linking monomer mixture (2-hydroxyethyl methacrylate (HEMA)/1,2-ethanediol dimethacrylate (EGDMA)) under similar reaction conditions. In general, transparency and softness of the gels decreased with increased use of water during the reaction, consistent with other literature reports [40]. Gels prepared with 1.5 mL of DI water were opaque and tougher compared to the other formulations and considered less suitable for forming flexible flow channels. In addition to mechanical flexibility, surface smoothness of the hydrogels was another key parameter in selecting a suitable material for fabricating the new 3D flow channels. pHEMA hydrogels synthesized with 1, 1.1, and 1.2 mL of DI water showed uneven surface textures owing to the increased softness of these materials. These formulations were not used for the flow channels as their rough surfaces could lead to friction artifacts in the flow of NPs.

**Figure 2:**
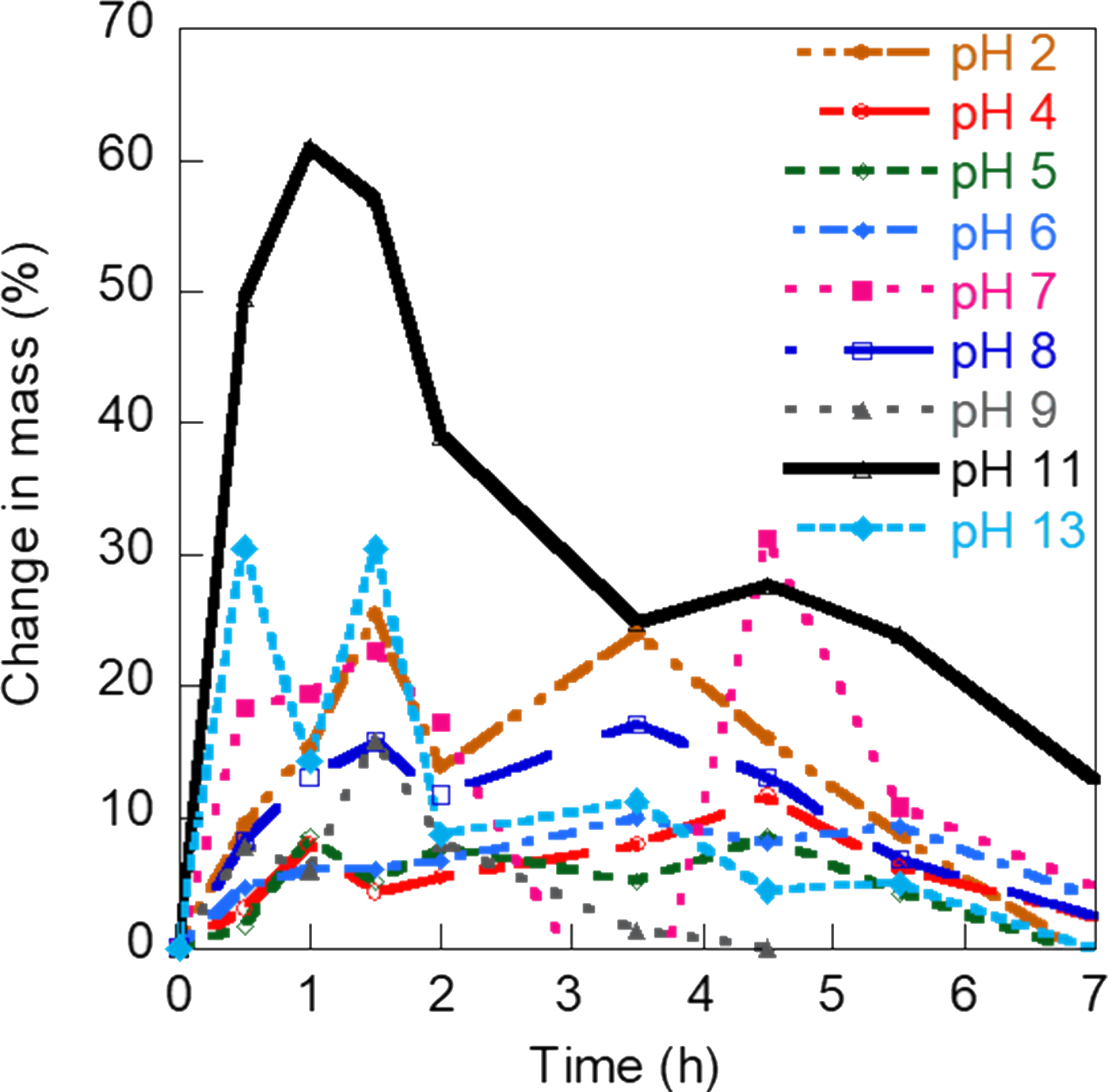
Plot showing pH-dependent swelling behavior of the pHEMA hydrogel prepared with 1.3 mL DI water over a period of 7 h.

The pHEMA hydrogel prepared with 1.3 mL of DI water was considered the most suitable formulation, in terms of mechanical flexibility, mechanical strength, and surface smoothness, for mimicking physiologically relevant microenvironments. This hydrogel also showed the highest capacity to retain water within its structure with a 60% increase in mass from swelling after 1 h of water absorption at pH 11 at ambient temperature (25 °C). The high water content of the pHEMA hydrogels is attractive as it resembles properties of soft tissues and vascular networks. Figure 2 shows the pH- and time-dependent swelling behavior of the pHEMA hydrogels selected for fabrication of the flow channels. In general, the different pHEMA gels showed maximum swelling after 1.5 h and 3.5 h with a 40–50% increase in mass and disintegrated after 7 h of water absorption (Figures S1–S3, Supporting Information File 1). The swelling behavior was highly influenced by the pH value of the solution. The selected gel formulation synthesized with 1.3 mL DI water showed an increased mass of 25% at pH 2, 30% at pH 13, and a maximum of 60% at pH 11.

**Figure 3:**
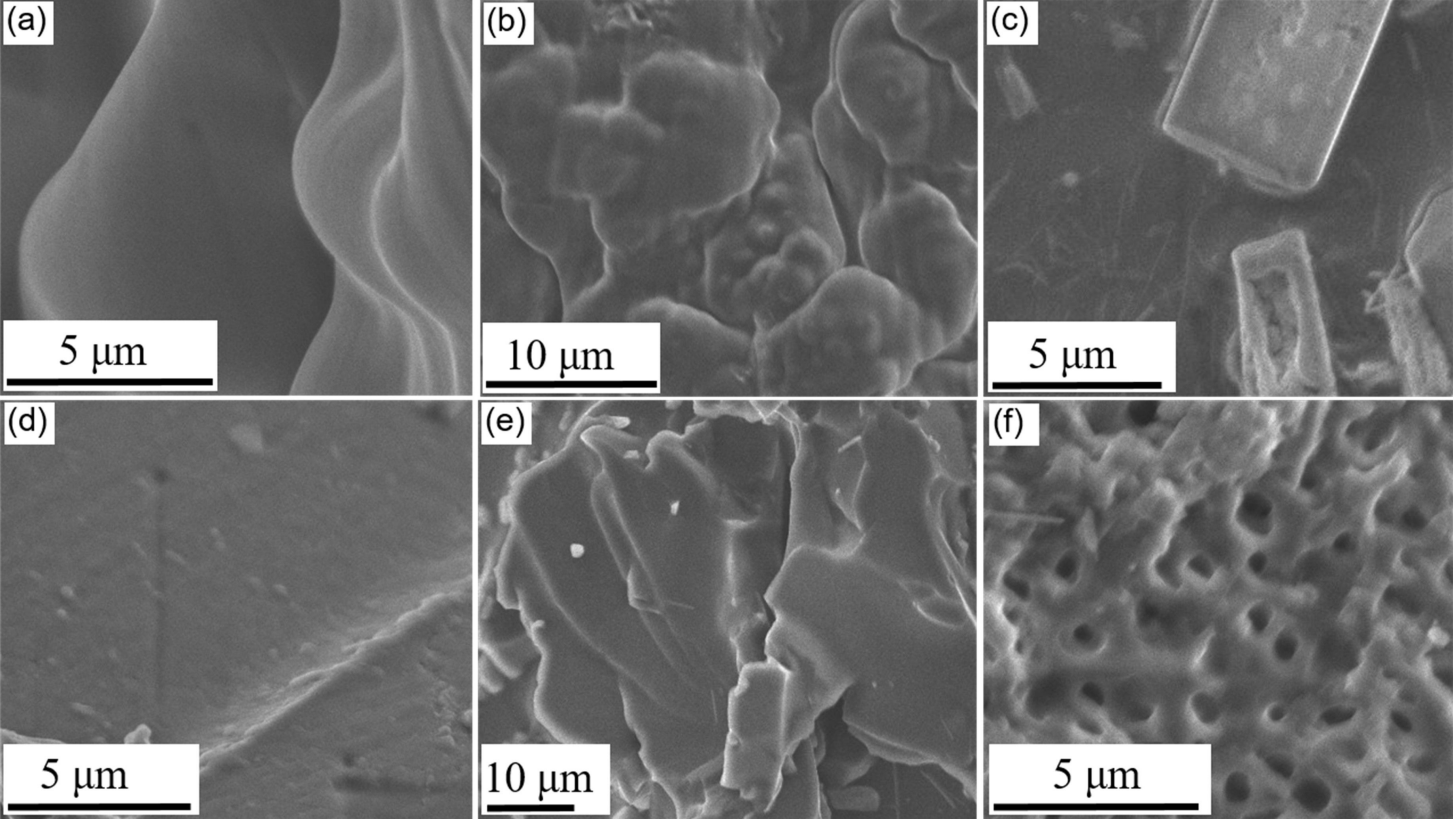
SEM images of pHEMA hydrogel samples synthesized using different quantities of DI water. (a) 1 mL, (b) 1.1 mL, (c) 1.2 mL, (d) 1.3 mL, (e) 1.4 mL, and (f) 1.5 mL.

Figure 3 shows the scanning electron microscopy (SEM) images of the different pHEMA hydrogels. The SEM characterization provided further insights into the variation of surface texture of the gels with the amount of reactant water used during synthesis. A soft, uneven, and layered texture was seen for the gels prepared with 1 mL DI water. The hydrogel prepared with 1.3 mL DI water showed the smoothest surface of all gel formulations. The gels prepared with 1.5 mL DI water showed a distinctly porous surface structure. Therefore, the 1.3 mL DI water hydrogels were most suitable for making flow channels of negligible friction to resemble the vascular microenvironment.

**Figure 4:**
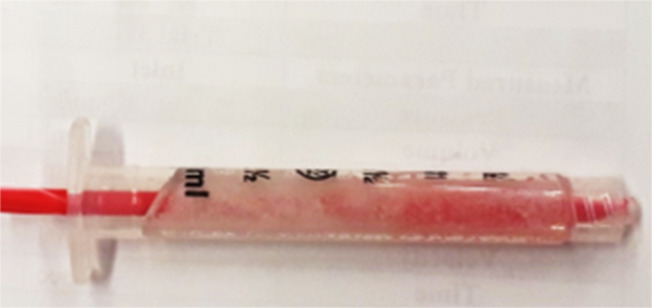
Facile syringe-tube assembly used in fabricating the hydrogel channels.

The hollow 3D hydrogel channels were formed via a facile experimental technique using a newly constructed syringe and plastic tube assembly (Figure 4). The liquid gel was allowed to solidify overnight within the syringe–tube assembly and slowly removed from the assembly after solidification. The resultant final product consisted of soft cylindrical channels with an internal diameter of 4 mm and a length of 47 mm. These dimensions of the conduits are comparable to the optimum vessel diameter reported by Bond et al. for designing a section of single vascular tube [41]. The hydrogel channels were subsequently used for studying the flow of NPs.

**Figure 5:**
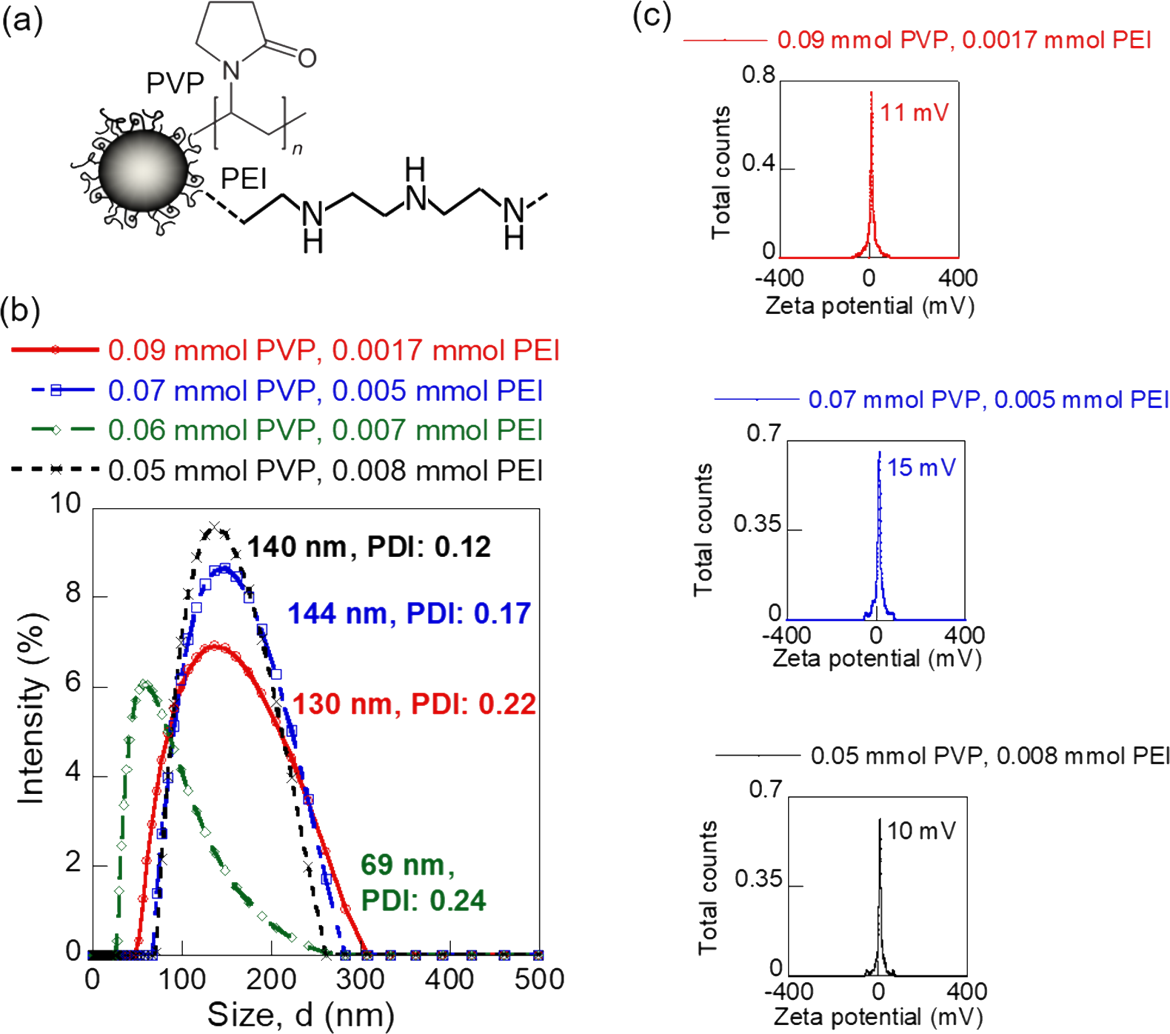
Different iron oxide NPs synthesized for the flow experiments. (a) Schematic overview, (b) hydrodynamic size plots, and (c) zeta potential plots.

Iron oxide NPs are extensively investigated in targeted therapy and drug delivery applications owing to their tunable size, surface functionalities, and magnetic properties. In this study, we synthesized four different polyvinylpyrrolidone (PVP)/polyethyleneimine (PEI)-coated iron oxide NPs (i.e., 0.09 mmol PVP/0.0017 mmol PEI, 0.07 mmol PVP/0.005 mmol PEI, 0.06 mmol PVP/0.007 mmol PEI, and 0.05 mmol PVP/0.008 mmol PEI) via a modified polyol method for understanding the flow of nanoscale drugs during drug delivery [34,42]. The different PVP/PEI ligand mixtures were used to obtain varying size and surface charge of the iron oxide NPs and to render the NPs biocompatible. Figure 5a shows a representative schematic of the iron oxide NPs synthesized. The hydrodynamic sizes and zeta potential values of the different iron oxide NPs were investigated in detail using a Litesizer 500 particle analyzer, before applying the aqueous NP solutions for flow experiments. All four types of the iron oxide NPs showed a monodisperse size distribution (Figure 5b). Among these, the iron oxide NPs prepared with 0.06 mmol PVP/0.007 mmol PEI showed the smallest size (69 nm) with a narrow polydispersity index (PDI) of 0.24. The NPs coated with 0.09 mmol PVP/0.0017 mmol PEI ligand mixture were 130 nm in size with a uniform size distribution (PDI: 0.22). In comparison, the 0.07 mmol PVP/0.005 mmol PEI-coated NPs and 0.05 mmol PVP/0.008 mmol PEI-coated NPs were slightly larger with sizes of 144 nm (PDI: 0.17) and 140 nm (PDI: 0.12), respectively. The iron oxide NP formulations showed similar surface charges (Figure 5c). The low absolute values of zeta potential of the NPs suggested positively charged surfaces stabilized via steric hindrances from the polymer coating. The experimental flow velocity and the mass loss during flow under a laminar flow regime were investigated for the four different NP sizes as a first step towards constructing an in vitro technique to complement the existing animal models for predicting flow and interaction of NP-based drugs in vivo.

**Figure 6:**
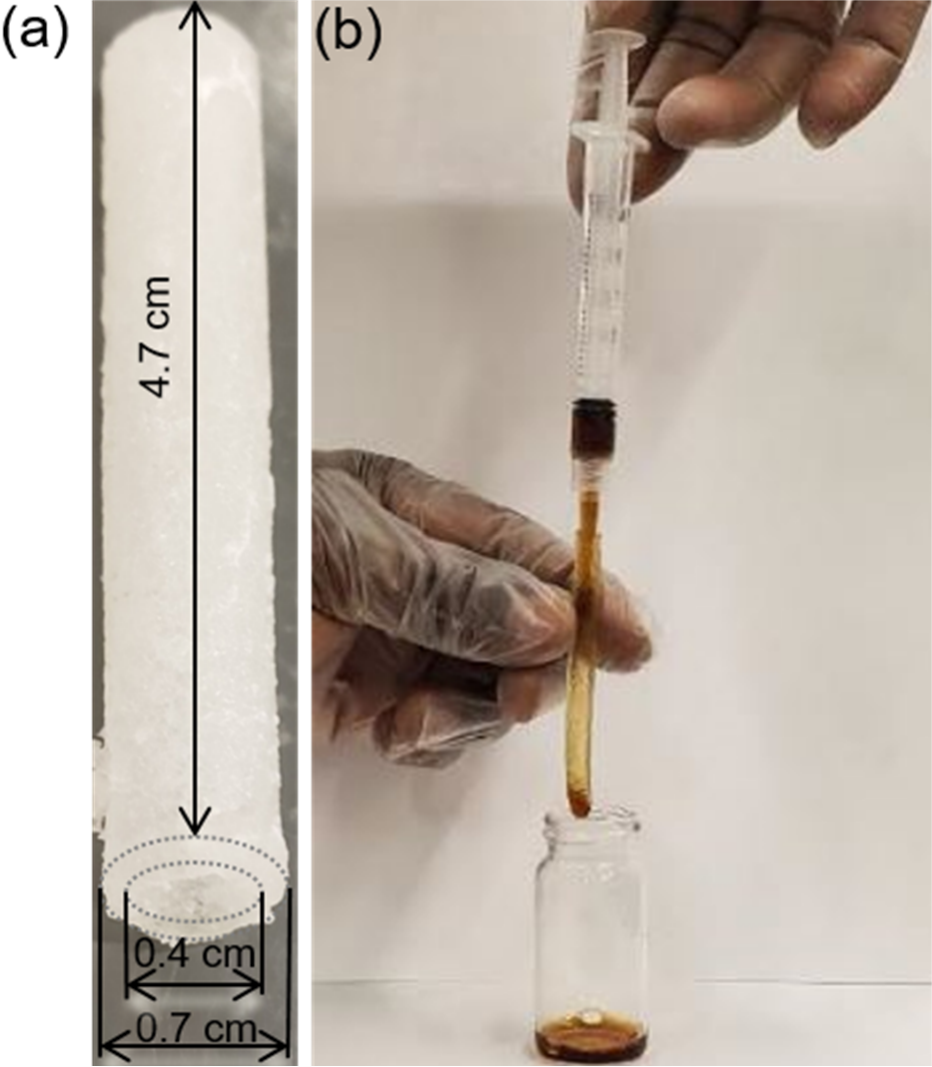
Experimental investigation of NP transport through soft hydrogel flow paths. (a) Representative hydrogel channel and (b) image showing a model flow experiment.

A typical flow experiment is shown in Figure 6. The iron oxide NPs were injected through the preformed pHEMA hydrogel channels using a syringe to facilitate a laminar flow. The injection of NPs was conducted manually as shown in Figure 6b in an effort to replicate clinical intravenous delivery conditions, keeping the syringe type and pressure similar for all experiments. Three consecutive experiments have been conducted for each flow condition as described in the experiments to account for variations in inlet pressure due to the manual delivery. Such manual injections have typically been used in drug delivery applications, but the variability in inlet force in this mode of delivery is currently leading to an increasing use of autoinjectors. In our future studies, we will use flow pumps to replicate the case of drug delivery using autoinjectors. The experimental flow profile of each NP was characterized over seven different mass concentrations (2.008–5.240 g) using two primary parameters as markers, the average flow velocity and mass loss of NPs over the flow path measured from the difference in inlet and outlet mass of the NP solutions.

**Figure 7:**
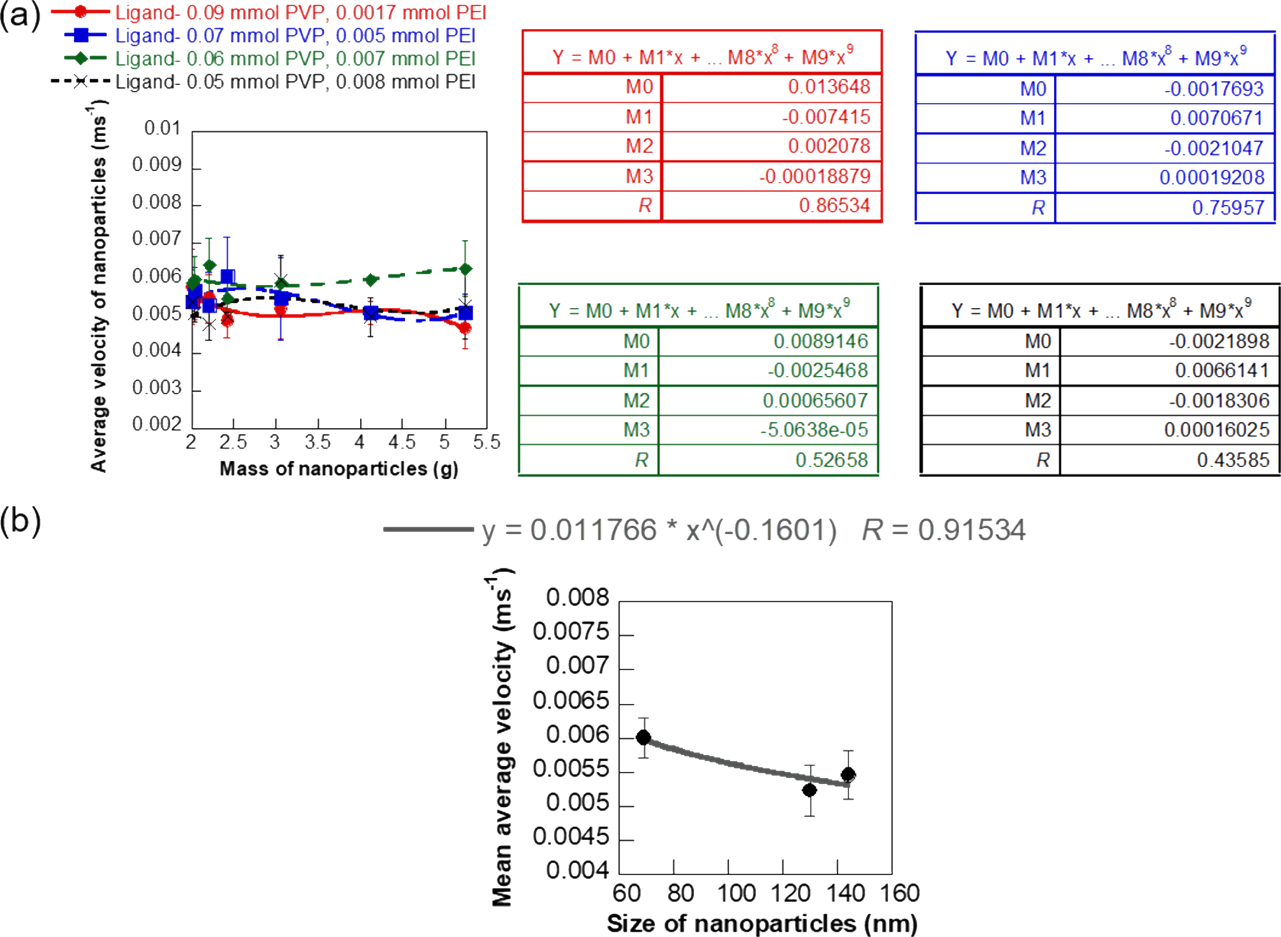
Experimental velocity profile of the iron oxide NPs. (a) Plot showing the average flow velocity of the different iron oxide NPs at different inlet mass concentrations and (b) variation of the average NP velocity with size of the NPs. The error bars represent standard deviation.

The experimental flow velocity of the four types of iron oxide NPs showed a polynomial variation with respect to NP mass during the transport through soft hydrogel channels (Figure 7a). The average velocity of 0.09 mmol PVP/0.0017 mmol PEI-coated NPs of size 130 nm ranged from 0.47 to 0.58 cm·s^−1^ and was a third-order polynomial function of the NP mass with an *R* value of 0.87. NPs coated with 0.07 mmol PVP/0.005 mmol PEI of size 144 nm exhibited a flow velocity ranging between 0.51 and 0.61 cm·s^−1^ with a cubic polynomial fit with respect to mass concentration of NPs (*R* = 0.76). The flow velocity of 0.06 mmol PVP/0.007 mmol PEI-coated iron oxide NPs of size 69 nm varied between 0.55 and 0.64 cm·s^−1^ while the 140 nm sized NPs with 0.05 mmol PVP/0.008 mmol PEI coating showed a velocity range of 0.48–0.6 cm·s^−1^. General cubic polynomial trends in velocity with respect to NP mass concentration were also observed for these two iron oxide NPs, but with higher deviations compared to the other NP formulations. The detailed experimental data for flow velocity helped us in determining size-dependent variations of the NP flow under physiologically relevant conditions mimicking vascular sections (Figure 7b). The average velocity of the different NPs as measured from seven different mass concentrations decreased with increase in size of the NPs following a power function trend with a reliable fit (*R* = 0.91). This trend in NP velocity is different from the flow velocity observed through plastic channels, indicating strong influence of the material of the flow channel in the flow trajectory of NPs. In the case of soft hydrogel-based flow channels constructed to mimic vascular networks, the larger sized NPs moved slower than the smaller NPs, similar to trends seen in macroscale objects. This phenomenon could be explained in terms of two factors, a dominance of Brownian forces over diffusion and hydrodynamic forces for the flow of these aqueous NP dispersions and additional frictional forces in the NP flow path due to the soft and uneven surface of the hydrogel channels. This size-dependent flow behavior of aqueous NP solutions within soft biomimetic channels is a key finding in terms of drug delivery applications as it can serve as an in vitro tool to predict the trajectory of NP drugs and their capacity to reach disease sites under clinical conditions. Specifically, the velocity of NPs in these experiments ranged from 0.47 to 0.64 cm·s^−1^, comparable to blood flowrates in capillaries, liver, or tumor. The blood flowrate vary in the range of 11–66 cm·s^−1^ for aorta, vena cava, and pulmonary arteries [43]. Klarhöfer et al. reported blood flowrates of 4.9–19 cm·s^−1^ in arteries and 1.5–7.1 cm·s^−1^ in veins of the human index finger [44]. Lower blood flowrates (0.08–0.25 cm·s^−1^) were recorded in capillaries [45]. Blood flow within the tumor and liver are also slower. This induces a fluid flow pattern from the center outwards in these regions and the accumulation of micro- and nanoparticles on the walls of the vasculature [46] leading to a possible loss of NP drugs.

**Figure 8:**
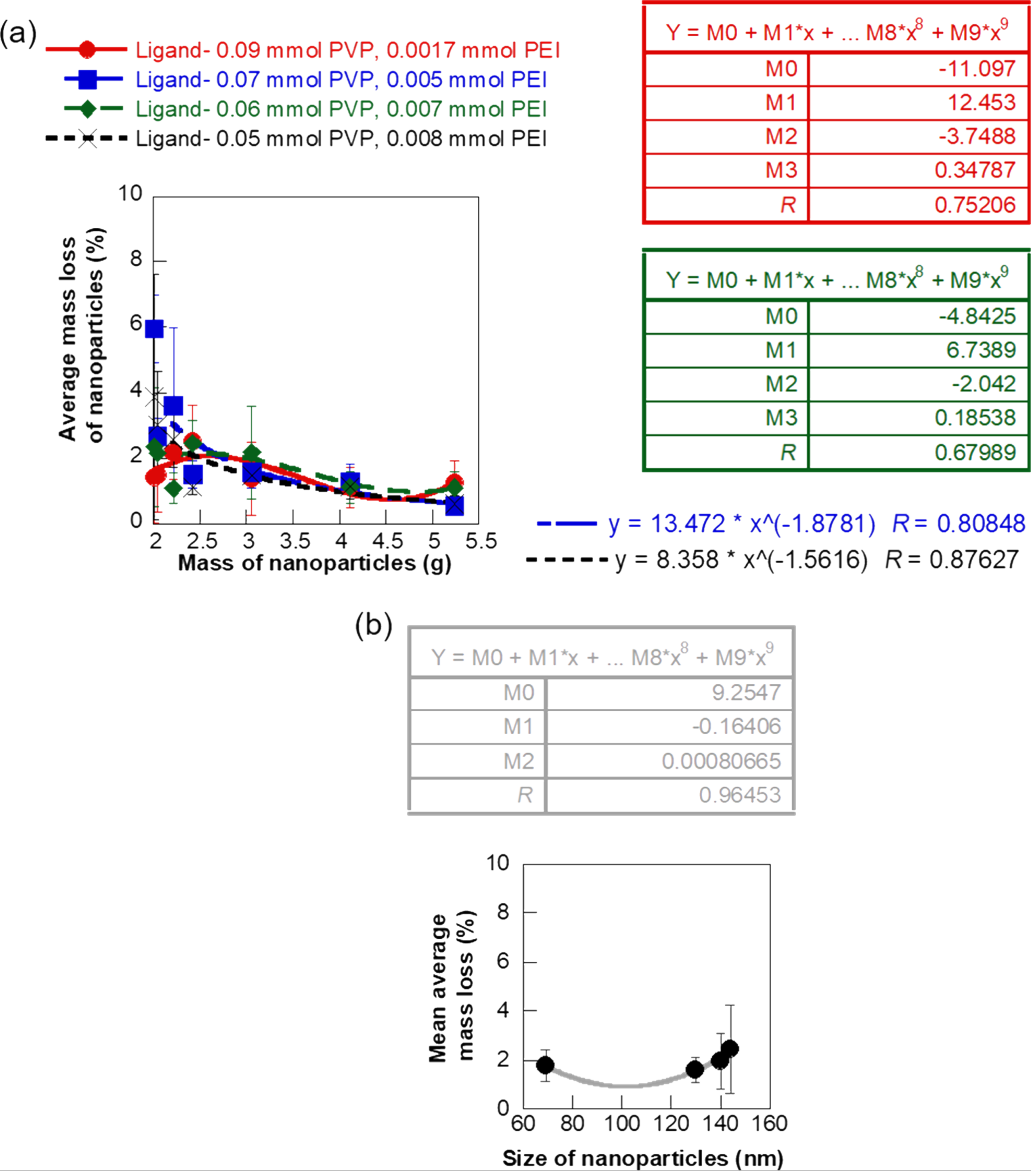
Experimental mass loss of the NPs during flow through hydrogel channels. (a) Plot showing average mass loss at different inlet mass concentrations of NPs and (b) variation in mass loss of NPs during flow with respect to size of the NPs. The error bars represent standard deviation.

Loss of the NP drug during transport to the disease site plays a major role in clinical efficiency of the drug. It is an important design consideration for engineering drug delivery systems. Therefore, it will be useful to be able to predict the quantity of NPs lost due to non-specific binding or deposition on the walls of the vascular network. In this study, we investigated the mass loss of the four different types of iron oxide NPs during flow through the soft biomimetic hydrogel channels by monitoring the mass of inlet and outlet solutions (Figure 8a). This experimental mass loss of the NPs in the flow path is also a measure of the NPs bound or deposited on the walls of the hydrogel channels under the specific flow conditions. Mass loss of the NPs was monitored for seven different inlet mass concentrations for each NP type to determine the concentration-dependent trend in NP binding or deposition during the flow. The 130 nm sized NPs coated with 0.09 mmol PVP/0.0017 mmol PEI showed a mass loss ranging between 1.10 and 2.50% with an average of 1.60% and a cubic polynomial trend in mass loss with respect to the inlet NP mass (*R* = 0.75). The mass loss of the 0.07 mmol PVP/0.005 mmol PEI ligand-coated iron oxide NPs, of size 144 nm, was also a cubic polynomial function of the inlet mass of the NPs, but a wider range of mass loss (0.53–5.96%, *R* = 0.68) was observed for these NPs with the highest average mass loss (2.45%) among the four types of NPs. In comparison, the smaller 69 nm sized NPs coated with 0.06 mmol PVP/0.007 mmol PEI showed a power function trend in mass loss with respect to the NP inlet mass concentration (*R* = 0.81). The mass loss varied between 1.09 and 2.45% with an average of 1.77% for these NPs. The 0.05 mmol PVP/0.008 mmol PEI ligand-coated iron oxide NPs, of size 140 nm, had a mass loss ranging from 0.60 to 3.87% with an average of 1.96% and a reliable power function trend with respect to the inlet mass (*R* = 0.88). Figure 8b shows the influence of NP size on the experimental mass loss from binding or deposition of the NPs during transport. The size-dependent trend in NP adhesion provides key insights into the mechanisms and dominating forces determining the flow trajectory of the NPs. The mass loss percentage of the iron oxide NPs increased with NP size following a reliable quadratic correlation (*R* = 0.96) in the case of flow through soft channels mimicking vascular constructs. This is similar to the NP mass loss due to binding or deposition observed in plastic flow channels. This general correlation between mass loss percentage and size of the NPs implies that smaller NPs, although having a larger relative surface area for binding show a lower affinity for binding or deposition and are more likely to reach the target diseased site. The lower binding affinity of the smaller NPs could be due to the Brownian motion of the particles and the influence of other surrounding particles and fluid. However, it is important to take a closer look at the correlation between mass loss of NPs and size for specific NP mass concentrations to better understand the different forces dominating the flow of NPs.

**Figure 9:**
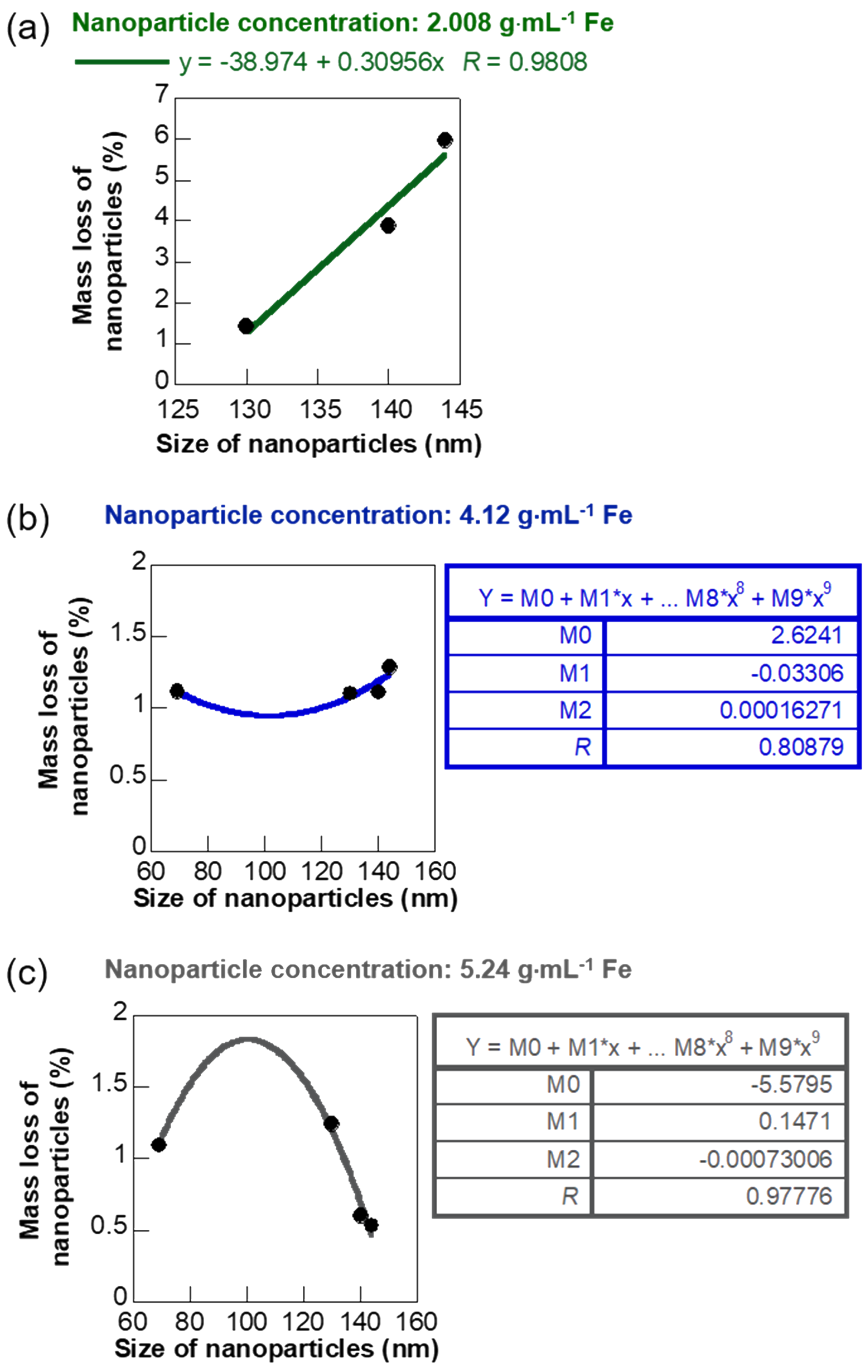
Plot showing the average mass loss percentage of NPs as a function of the size at three different inlet mass concentrations. (a) 2.008 g·mL^−1^ Fe, (b) 4.12 g·mL^−1^ Fe, and (c) 5.24 g·mL^−1^ Fe.

Figure 9 shows the variation of average mass loss percentage of the NPs as a function of the NP size for three specific inlet mass concentrations of the NPs. The mass loss of NPs during flow through the soft hydrogel channels is primarily caused by the deposition or binding of the NPs to the walls of the channels. Therefore, the mass loss correlations provide experimental insights into the concentration-dependent changes in the binding affinity of NPs that lead us to the mechanisms dominating the NP flow. The mass loss percentage and hence the binding of the NPs increased with NP size following a reliable linear correlation (*R* = 0.98) for the lowest inlet mass concentration of the NPs (2.008 g Fe). This implied that the smaller NPs, despite having a higher relative surface area had less tendency to deposit or bind at the lower concentrations. Therefore, the NP flow was likely dominated by the velocity of the surrounding fluid and Brownian motion at these concentrations, which prevented the deposition of the lighter, small-sized NPs [18,47–48]. In comparison, the larger NPs had more time for interaction and binding to the walls of the channel as they moved with a slower velocity through the hydrogel flow path. An increase in mass loss with size of NPs was also observed for a higher NP concentration of 4.12 g·mL^−1^ Fe, but the variation was more gradual. This phenomenon suggested a slightly lower influence of the surrounding fluid and Brownian motion on the NP flow in this concentration range. However, the quantity of NPs lost due to binding or deposition showed a decreasing quadratic correlation with NP size at the highest inlet mass concentration (5.24 g Fe). This phenomenon showed a dominance of particle properties and diffusion-dominated binding and deposition at the highest concentration. Recently, Liu et al. and Decuzzi et al. reported computational models predicting a similar inverse correlation of NP adhesion with size in diffusion-driven flow [18,49]. Thus, the experimental mass loss trend at different concentrations helped us in understanding the different influences on NP flow at these regimes. We further enhanced the reliability of our approach to predict NP flow through physiologically relevant environments by coupling CFD simulation with the experimental results.

**Figure 10:**
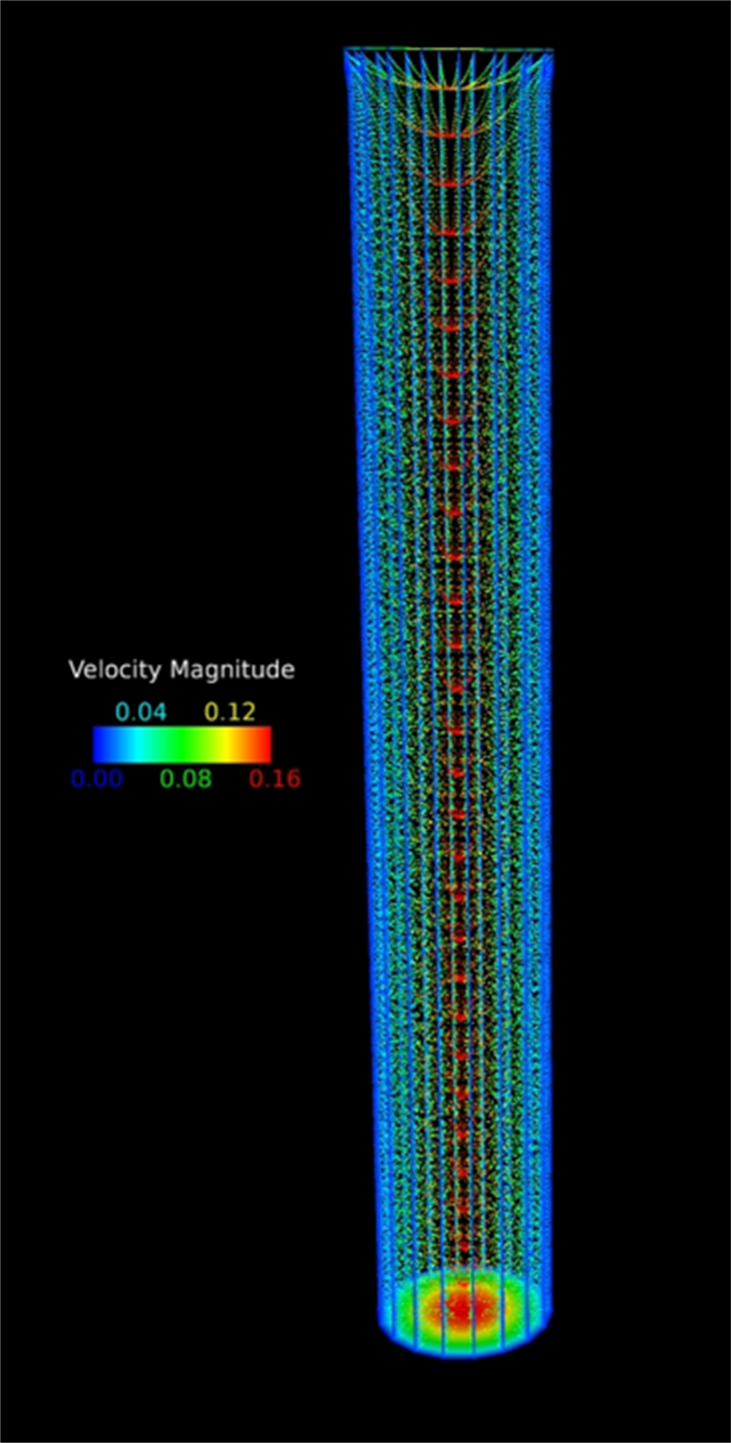
Velocity profile of NPs flowing through the hydrogel channel using CFD.

A computational analysis was conducted for nanofluid flow through an unbent cylindrical hydrogel channel to further confirm the applicability and accuracy of our experimental results. Two aqueous dispersions of 0.07 mmol PVP/0.005 mmol PEI-coated iron oxide NPs (size: 144 nm) of inlet mass concentrations of 4.12 g and 2.008 g and density of 5.24 g·mL^−1^ were investigated in these CFD simulations. A pointwise mesh generation software was used to generate the geometry and grid for simulating the nanofluid flow. Meshing, typically involves both surface and volume meshing and the generated surface grids greatly affect the quality of volume grids. Therefore, a finer grid scheme was required in the boundary layer to obtain an accurate flow field near the hydrogel channel. The first grid point spacing nearest the wall was 3.0 × 10^−4^ to achieve a desired value of y+ ≤ 1 value. Since the geometry is an unbent circular tube, the structured mesh is found suitable for the better convergence of simulations. A grid refinement study was made with several different structured meshes in each coordinate direction. The effect of altering the cell size was investigated with grids of 65, 129 and 193 cells in the stream-wise direction and 25, 49 and 97 cells in the radial direction. The computation began with a coarse grid (65 × 25 × 37) and was gradually refined in each coordinate direction until the changes observed in the solutions were insignificant. In this study, grid independency study showed that after the grid quantity reached a certain quantity, a larger number of grids did not affect the accuracy. 462,981 cells (129 × 97 × 37) was found to be the optimum mesh size. This was used as the grid number in our case with inlet, outlet, and no-slip wall of the hydrogel channel as the boundary conditions for the geometry. The nanofluid entered the hydrogel channel with an average velocity of 0.0051 m·s^−1^ and 0.0054 m·s^−1^ for which the Reynolds numbers based on the diameter of the tube were 120.1 and 127.1, respectively. The fluid flow within the hydrogel channel was considered laminar and Newtonian. Particle–particle interactions between erythrocytes and NPs and ligand–receptor binding have not been considered in this model as we first wanted to understand the flow of NPs through soft, biomimetic channels. In addition, particles that came in contact with the wall were assumed to be deposited. Figure 10 shows a representative result of the simulated laminar viscous flow through the hydrogel channel with fully-developed laminar entry flow. The results suggested that the NPs followed the streamlines of the flow field for this laminar flow regime. The computed pathlines, colored by velocity magnitude to visualize the NP flow for the total inlet mass of 4.12 g is presented in Figure 10. The mass losses computed on the refined meshes did not appreciably vary from those computed on the corresponding previous meshes (129 × 49 × 37 and 193 × 97 × 37), and it was safely assumed that the simulation results are grid independent. The computed mass losses of the particles are 1.341% and 6.253% for 4.12 g and 2.008 g inlet concentrations of NP fluid flow in hydrogel channels, respectively. It is observed that the numerical simulation results favorably agree with experimental data (1.286% and 5.96%) collected from the physical model that is reported in the manuscript.

## Conclusion

We developed a new in vitro technique coupling experimental flow studies and CFD analysis for predicting the velocity and binding affinity of the NPs through soft channels mimicking vascular networks. The biomimetic flow channels were synthesized via a new and facile fabrication route using pHEMA hydrogels. We also formed four different size and surface-controlled iron oxide NPs to serve as model nanoscale agents in the experimental analysis of NP flow under physiologically relevant conditions. These engineered experiments for understanding the mechanisms of NP transport in physiological microenvironments with pHEMA hydrogel channels are the first of its kind, to the best of our knowledge. We discovered that the flow trajectory of aqueous NP dispersions was greatly influenced by the Brownian forces, surrounding fluid, and the material of the flow channel at the lower mass concentrations, based on the flow experiments conducted at seven different concentrations for each of the four NP formulations. The NPs showed a diffusion-dominated trend in flow at the highest concentration. These results were in close confirmation with our CFD model for a fully developed laminar flow of NPs through the cylindrical hydrogel constructs. Therefore, this new in vitro method for predicting NP flow is a combination of both experimental and computational techniques targeted at practically relevant flow conditions for drug delivery. The reported in vitro approach will be highly relevant at the preclinical stages to catalyze higher clinical success rates for drug delivery systems using NPs. Future efforts will be directed at synthesizing hydrogel flow channels with wider ranges of orifice size similar to the human vascular network for further validating the practical applicability of our in vitro NP flow analysis.

## Experimental

### Chemicals used

Reagents including 2-hydroxyethyl methacrylate (HEMA, Sigma-Aldrich, 97%), 1,2-ethanediol dimethacrylate (EGDMA, Sigma-Aldrich, 98%), ammonium persulfate ((NH_4_)_2_S_2_O_8_, AmPS, Sigma-Aldrich, 98%), sodium metabisulfite (Na_2_S_2_O_5_, NaMBS, Fisher, 99.4%), inhibitor remover (Sigma-Aldrich), iron(III)-2,4-pentanedionate (Fe(acac)_3_, Alfa Aesar), polyethyleneimine (PEI, Alfa Aesar, 60 kDa – 50 wt % aqueous solution), polyvinylpyrrolidone (PVP, TCI, 10 kDa, 99%), triethylene glycol (TREG, ACROS, 99%), sodium hydroxide (NaOH, Fisher, 99%), hydrochloric acid (HCl, Carolina Biological Supply, 6 M), and deionized water (DI, Fisher) were purchased from Sigma-Aldrich and Fisher and used without further purification.

### Synthesis of pHEMA hydrogels

pHEMA hydrogels were formed via chemical cross-linking of two hydrophilic monomers, HEMA and EGDMA in a room temperature synthesis. Six different biocompatible pHEMA hydrogels were synthesized in this study by varying the quantity of DI water used during preparation of the hydrogels, keeping all the other parameters constant. In a typical synthesis, the EGDMA cross-linker was added at an amount of 3 mol % HEMA to six different aqueous solutions of HEMA (38.4 mmol, 5 g) prepared using different quantities of DI water (1–1.5 mL). The HEMA/EGDMA solutions were first mixed well via sonication (Branson 1800, Fisher) at room temperature for one hour. Then, the redox initiators, NaMBS and AmPS were added to the mixtures at a quantity of 1 wt % relative to HEMA. The solutions containing monomers, cross-linkers, and the initiators were stirred at room temperature for 2 h in plastic vials. Finally, the solutions were left to solidify under the chemical hood for 12 h to form the different hydrogels. The hydrogels were subsequently characterized via swelling studies and SEM to find the best suitable soft material for fabrication of flow paths that mimic physiologically relevant properties in terms of flexibility and smoothness.

### Synthesis of iron oxide nanoparticles

In a manner closely related to the method in [34], four different iron oxide NP formulations were synthesized as models for NP-based drugs in this study to understand their flow behavior for drug delivery applications. A modified polyol method was used for the synthesis of the NPs. In a typical synthesis, the ligand mixture of PVP and PEI was heated to dissolution in the solvent TREG at 90 °C for 10 min. The iron precursor, Fe(acac)_3_ was subsequently added to this reactant mixture and thermally decomposed at 290 °C for 1 h to form the iron oxide NPs. The different biocompatible iron oxide NP formulations were synthesized by varying the PVP/PEI ligand mixture (e.g., 0.09 mmol PVP/0.0017 mmol PEI, 0.07 mmol PVP/0.005 mmol PEI, 0.06 mmol PVP/0.007 mmol PEI, and 0.05 mmol PVP/0.008 mmol PEI), keeping all other parameters the same. All syntheses were conducted in a N_2_ atmosphere inside a Schlenk line setup. The NP product was directly soluble in water and was further purified via high-speed centrifugation (Fisher Scientific) at 14000 rpm for 30 min to remove the excess organics as supernatants from the NP precipitates. The cleaning via centrifugation was conducted two times and the remaining NP precipitate was re-dissolved in DI water to obtain the final NP product of target concentration. Seven different concentrations ranging from 0.011 to 11.07 g·mL^−1^ were prepared for each type of iron oxide NPs in this study. All NP samples were mixed well via a 30 min sonication (Branson 1800, Fisher) at room temperature for subsequent use in the transport experiments.

### Characterization

#### Characterization of hydrogels

The morphology and texture of the different hydrogels were characterized on a Hitachi S3400 SEM. Hydrogel samples for SEM imaging were prepared by cutting thin cross-sectional slices of 15 mm diameter. The samples were subsequently loaded on to the sample holder for SEM viewing.

The swelling behavior of each pHEMA hydrogel was investigated at 25 °C over a period of two days for pH values ranging from 1 to 13. Aqueous NaOH or HCl was mixed with DI water dropwise to obtain the desired pH values for the swelling characterization of the gels. A section of the respective hydrogel of weight 8 mg was cut and used as the sample for the respective swelling study. The initial weight (*W*_dry_) of each gel was measured after drying the gels at room temperature under vacuum for three days. Each gel type was immersed in 13 different solutions (5 mL, pH 1–13) and the weights of the swollen hydrogels (*W*_swollen_) were recorded at regular intervals after carefully removing the hydrogel from the solution and blotting the surface water with filter paper. The extent of swelling of the hydrogel (*W*_t_) was measured using the following equation:


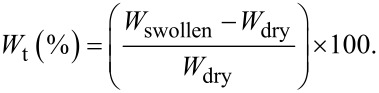


All measurements of weight and degree of swelling of the hydrogels were reported as an average of three swelling studies. Finally, the dynamic swelling behavior of different pHEMA hydrogels synthesized in this study were graphically summarized using 95% student’s *t* confidence interval as error bars. A combination of swelling behavior results and surface texture characterization of the gels via SEM imaging was used to select the best suitable hydrogel formulation to fabricate the flow channels mimicking physiological structures for the subsequent transport studies.

#### Characterization of iron oxide nanoparticles

A Litesizer 500 (Anton Paar) particle analyzer equipped with zeta potential capability was used to measure the hydrodynamic size, stability, and surface charge of aqueous dispersions of iron oxide NPs at pH 7 at room temperature (Figure S4, Supporting Information File 1). The aqueous NP samples were well dispersed via sonication for 15 min (Branson 1800, room temperature) prior to the measurements for representative results. The hydrodynamic sizes of each iron oxide NP sample were obtained as an average of three consecutive measurements for reliability. The zeta potential measurements were conducted at 25 °C using Omega cuvettes. Results for each NP sample were reported based on an average of four consecutive measurements.

### Fabrication of hydrogel channels

3D flow channels were engineered using the chemically cross-linked pHEMA hydrogel formulation prepared with 1.3 mL DI water. A plastic syringe and tube assembly was constructed to fabricate the cylindrical hydrogel flowpath. The liquid pHEMA hydrogel mixture was transferred to a 3 mL plastic syringe (Fisher). The thin plastic tube of diameter 4 mm was inserted through the open end of the syringe to form the internal perforation and path for the flow of NPs. The hydrogel was left to solidify for 12 h within the syringe and tube assembly. The soft three-dimensional hydrogel structure was then removed from the syringe to form the channels for subsequent flow studies with iron oxide NPs.

### Transport experiments

The flow studies were conducted with four different iron oxide NPs as model drug delivery agents and pHEMA hydrogel channels as model biomimetic vascular structures. Seven different mass concentrations ranging from 2.008 to 5.24 g were used for each NP type to experimentally investigate the velocity and mass loss of NPs during the flow. Tables S1–S4 (Supporting Information File 1) summarize the experimental conditions and results from the flow of NPs through biomimetic hydrogel channels. In a typical transport study, 1 mL aqueous dispersion of NPs was injected via a syringe for vertical flow through the straight hydrogel channel and collected in a vial at the end of the flow path. The mass of NPs was recorded both at the inlet and outlet of the flow path to determine the loss of NPs due to adherence to the walls of the channel. The mass loss of NPs for each flow experiment was measured as a difference of the mass of NPs injected at the inlet of the hydrogel channel and the mass of NPs collected at the outlet. Each experiment for a specific concentration of the NP type was repeated three times, based on which the average mass loss was reported for the specific case. The time taken for the NPs to flow through the length of the channel was recorded for subsequent measurement of velocity of the NPs. All experiments were conducted at room temperature using similar flowpaths constructed with the same formulation of pHEMA hydrogel for consistency.

### Computational methods

CFD simulations for nanofluid flow and mass loss of NPs in flow through hydrogel channels were conducted via a previously reported method using our in-house Tenasi flow solver [34,50–51]. Table S5 and Table S6 in Supporting Information File 1 summarize some of the key parameters used for CFD analysis of NP flow through hydrogel channels.

## Supporting Information

The supporting information includes the following items: swelling studies of the different pHEMA hydrogel formulations, Tables S1–S4 summarizing the experimental flow velocity and mass loss data for the four different iron oxide NPs, Table S5 and Table S6 describing the important parameters and NP properties used for the CFD simulations, and DLS size plot of representative iron oxide NP after prolonged storage at room temperature.

File 1 Additional experimental and computational data.
