# Supplementary material for: Understanding nanoparticle flow with a new in vitro experimental and computational approach using hydrogel channels
Source: Beilstein J Nanotechnol. 2020 Feb 6;11:296–309. doi: 10.3762/bjnano.11.22 (PMC7034222; doi:10.3762/bjnano.11.22)
Supplement: File 1 — Additional experimental and computational data. [file Beilstein_J_Nanotechnol-11-296-s001.pdf]

BEILSTEIN JOURNAL OF NANOTECHNOLOGY

Supporting Information

for

Understanding nanoparticle flow with a new in vitro experimental and computational approach using hydrogel channels

Armel Boutchuen, Dell Zimmerman, Abdollah Arabshahi, John Melnyczuk and Soubantika Palchoudhury

Beilstein J. Nanotechnol. **2020,** *11,* 296–309. doi:10.3762/bjnano.11.22

Additional experimental and computational data

License and Terms: This is a supporting information file under the terms of the Creative Commons Attribution License (https://creativecommons.org/licenses/by/4.0). Please note that the reuse, redistribution and reproduction in particular requires that the authors and source are credited.
The license is subject to the *Beilstein Journal of Nanotechnology* terms and conditions: (https://www.beilstein-journals.org/bjnano)

Figures S1–S3 show the swelling studies of poly(hydroxyethyl)methacrylate (pHEMA) hydrogels synthesized with different quantities of DI water. The error bars represent deviations based on 95% confidence intervals.

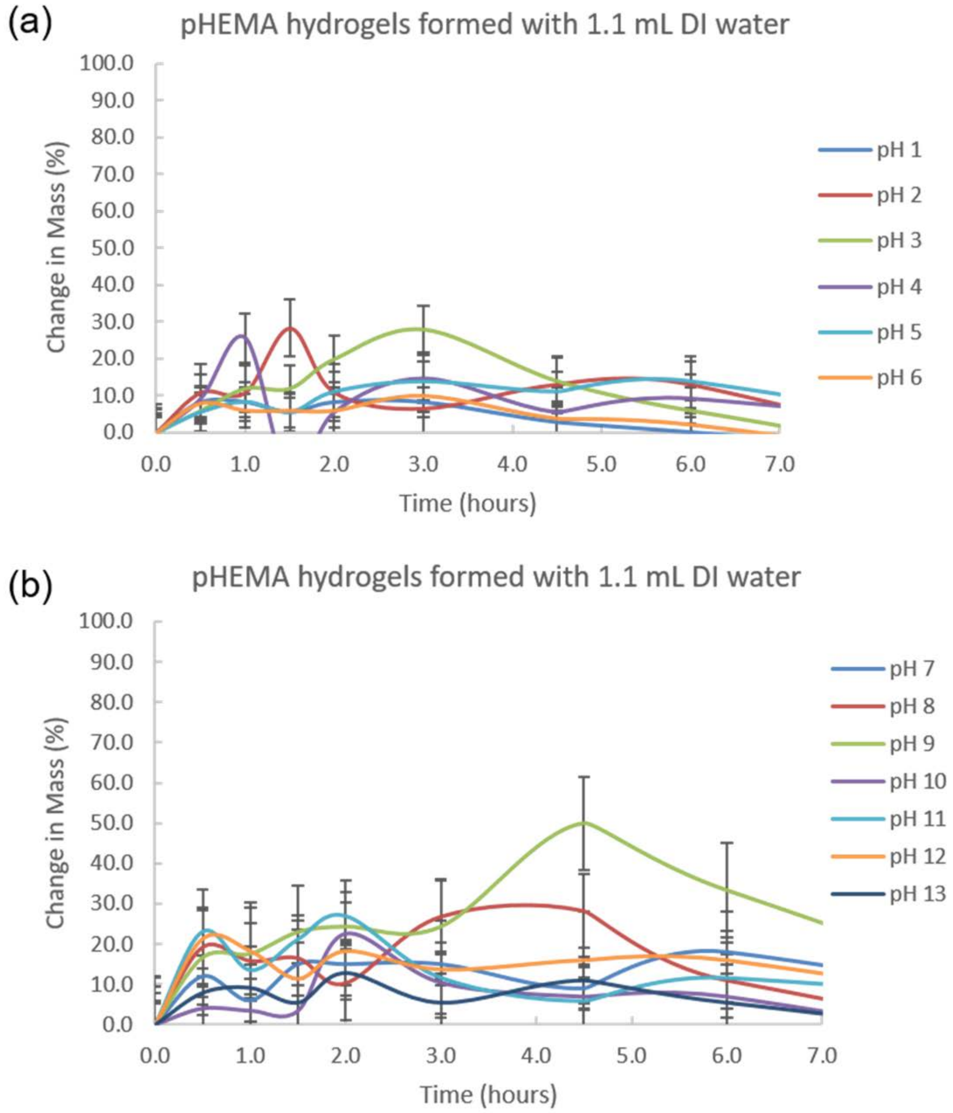

Figure S1: pH-Dependent swelling studies of pHEMA hydrogels synthesized with 1.1 mL DI water.

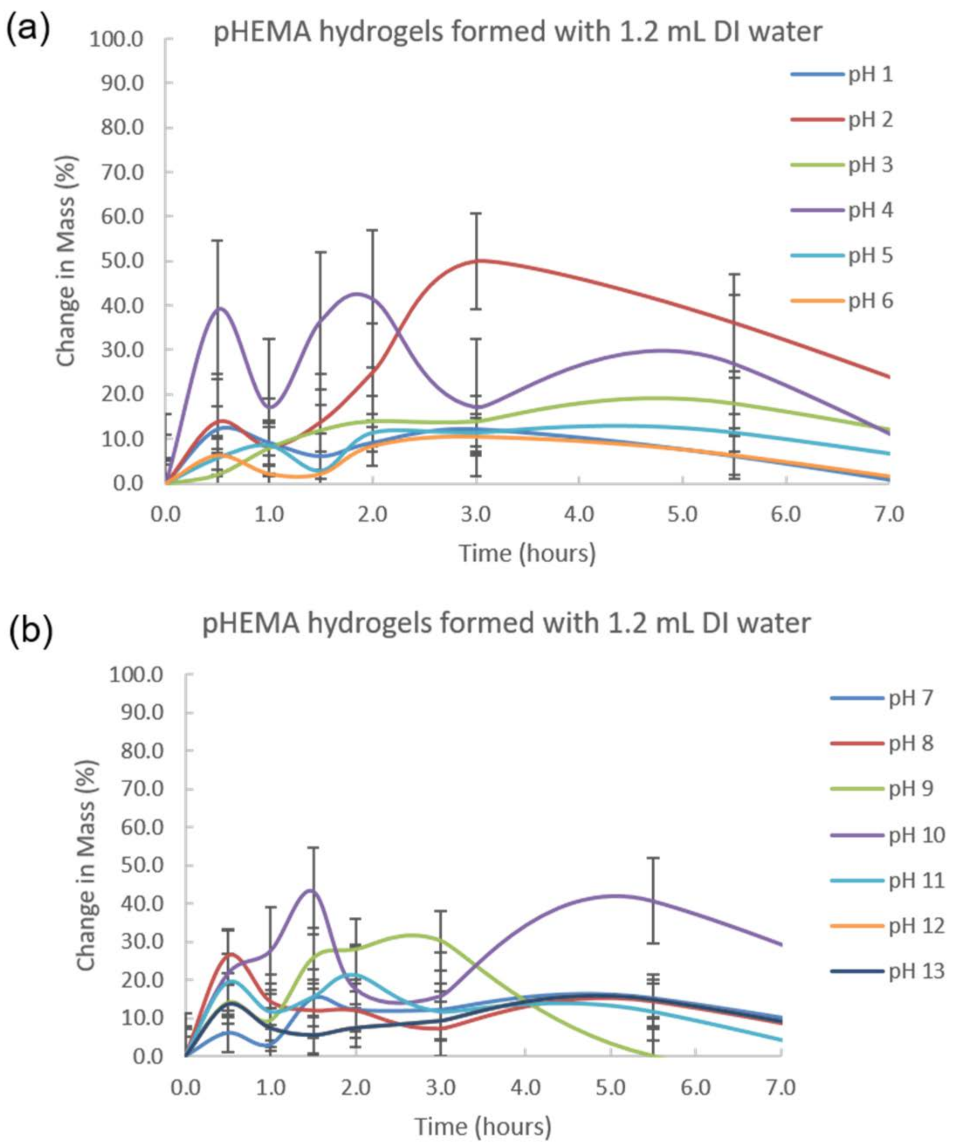

Figure S2: pH-Dependent swelling studies of pHEMA hydrogels synthesized with 1.2 mL DI water.

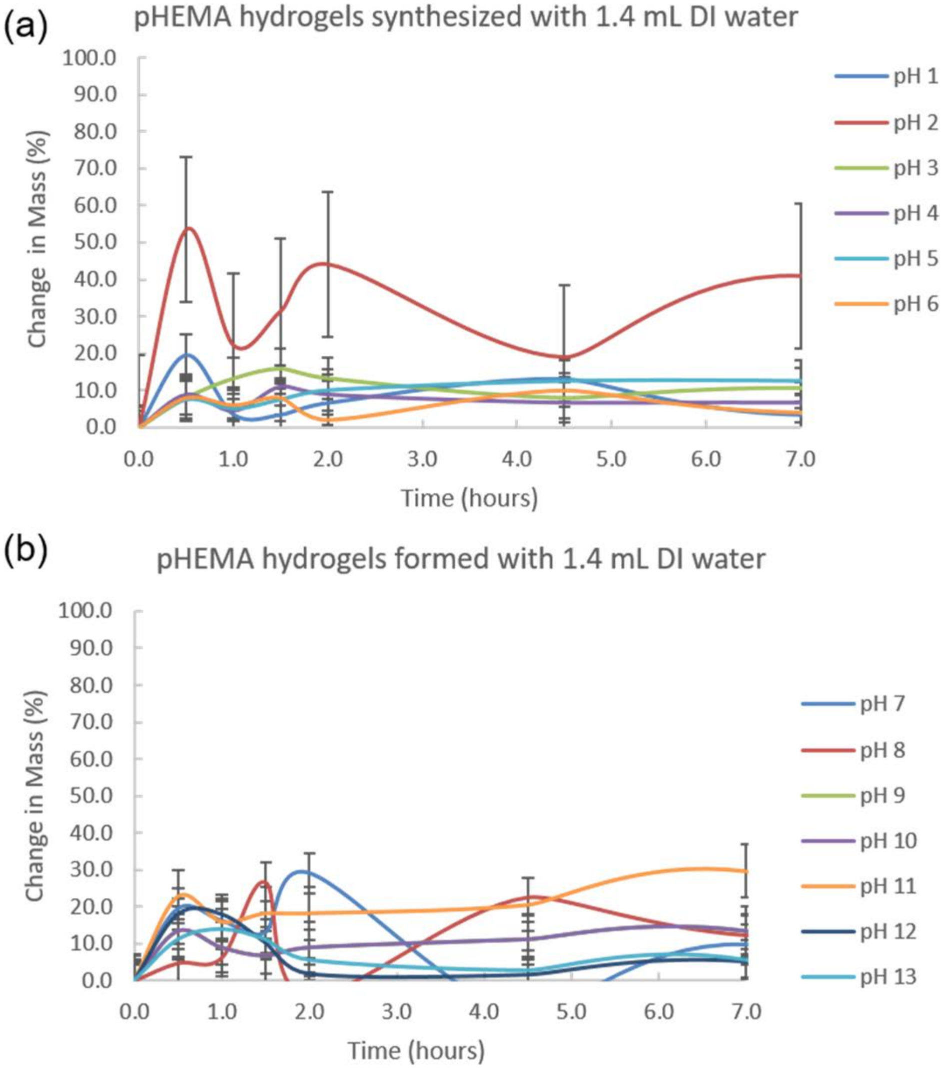

Figure S3: pH-dependent swelling studies of pHEMA hydrogels synthesized with 1.4 mL DI water.

Tables S1–S4 highlight the results from the novel experimental flow studies of different iron oxide nanoparticles (NPs) through hydrogel channels.

Table S1: Results from experimental flow of 0.09 mmol PVP/0.0017 mmol PEI-coated iron oxide nanoparticles (hydrodynamic diameter ~ 130 nm, PDI: 0.22, zeta potential: 11 mV) through hydrogel channels.

No	Nanoparticle concentration (mg/mL Fe)	Mass of nanoparticles (g)	Trial No.	Pressure at inlet (atm)	Inlet volume (mL)	Pressure at outlet (atm)	Mass loss due to adhesion (g)	Average mass loss (g)	% mass loss (Avg)	Time elapsed (s)	Velocity (m/s)	Average velocity (m/s)
1	0.011	2.008	1	1.6	1	1	0.058	0.028	1.411	7.36	0.0061	0.0058
2	0.011	2.008	2	1.6	1	1	0.003	0.028	1.411	9.65	0.0047	0.0058
3	0.011	2.008	3	1.6	1	1	0.024	0.028	1.411	6.82	0.0066	0.0058
4	0.056	2.042	1	1.6	1	1	0.054	0.030	1.453	9.32	0.0048	0.0054
5	0.056	2.042	2	1.6	1	1	0.026	0.03	1.453	8.01	0.0056	0.0054
6	0.056	2.042	3	1.6	1	1	0.009	0.03	1.453	7.93	0.0057	0.0054
7	0.284	2.212	1	1.6	1	1	0.064	0.048	2.155	9.12	0.0049	0.0055
8	0.284	2.212	2	1.6	1	1	0.051	0.048	2.155	7.26	0.0062	0.0055
9	0.284	2.212	3	1.6	1	1	0.028	0.048	2.155	8.56	0.0053	0.0055
10	0.583	2.424	1	1.6	1	1	0.084	0.061	2.503	10.1	0.0045	0.0049
11	0.583	2.424	2	1.6	1	1	0.031	0.061	2.503	9.25	0.0049	0.0049
12	0.583	2.424	3	1.6	1	1	0.067	0.061	2.503	8.43	0.0053	0.0049
13	1.581	3.060	1	1.6	1	1	0.081	0.042	1.362	9.8	0.0046	0.0052
14	1.581	3.060	2	1.6	1	1	0.017	0.042	1.362	9.23	0.0049	0.0052
15	1.581	3.060	3	1.6	1	1	0.027	0.042	1.362	7.39	0.0061	0.0052
16	3.690	4.120	1	1.6	1	1	0.075	0.045	1.1	8.83	0.0051	0.0051
17	3.690	4.120	2	1.6	1	1	0.028	0.045	1.1	9.42	0.0048	0.0051
18	3.690	4.120	3	1.6	1	1	0.033	0.045	1.1	8.35	0.0054	0.0051
19	11.070	5.240	1	1.6	1	1	0.101	0.065	1.24	8.4	0.0054	0.0047
20	11.070	5.240	2	1.6	1	1	0.063	0.065	1.24	10.12	0.0044	0.0047
21	11.070	5.240	3	1.6	1	1	0.031	0.065	1.24	10.25	0.0044	0.0047

Table S2: Results from experimental flow of 0.07 mmol PVP/ 0.005 mmol PEI-coated iron oxide nanoparticles (hydrodynamic size ~ 144 nm, PDI: 0.17, zeta potential: 15 mV) through hydrogel channels.

No	Nanoparticle concentration (mg/mL Fe)	Mass of nanoparticles (g)	Trial No.	Pressure at inlet (atm)	Inlet volume (mL)	Pressure at outlet (atm)	Mass loss due to adhesion (g)	Average mass loss (g)	% mass loss (Avg)	Time elapsed (s)	Velocity (m/s)	Average velocity (m/s)
1	0.011	2.008	1	1.6	1	1	0.103	0.120	5.958	9.49	0.0057	0.0054
2	0.011	2.008	2	1.6	1	1	0.113	0.12	5.958	10.96	0.0049	0.0054
3	0.011	2.008	3	1.6	1	1	0.143	0.12	5.958	9.49	0.0057	0.0054
4	0.056	2.042	1	1.6	1	1	0.06	0.054	2.660	10.91	0.0049	0.0057
5	0.056	2.042	2	1.6	1	1	0.041	0.054	2.66	9.15	0.0059	0.0057
6	0.056	2.042	3	1.6	1	1	0.062	0.054	2.66	8.76	0.0062	0.0057
7	0.284	2.212	1	1.6	1	1	0.139	0.080	3.617	12.28	0.0044	0.0053
8	0.284	2.212	2	1.6	1	1	0.061	0.08	3.617	9.94	0.0054	0.0053
9	0.284	2.212	3	1.6	1	1	0.04	0.08	3.617	8.7	0.0062	0.0053
10	0.583	2.424	1	1.6	1	1	0.043	0.037	1.513	7.59	0.0071	0.0061
11	0.583	2.424	2	1.6	1	1	0.025	0.037	1.513	10.8	0.0050	0.0061
12	0.583	2.424	3	1.6	1	1	0.042	0.037	1.513	8.63	0.0063	0.0061
13	1.581	3.060	1	1.6	1	1	0.064	0.048	1.558	11.19	0.0048	0.0055
14	1.581	3.060	2	1.6	1	1	0.039	0.048	1.558	7.97	0.0068	0.0055
15	1.581	3.060	3	1.6	1	1	0.04	0.048	1.558	11.11	0.0049	0.0055
16	3.690	4.120	1	1.6	1	1	0.06	0.053	1.286	10.71	0.0050	0.0051
17	3.690	4.120	2	1.6	1	1	0.029	0.053	1.286	10.52	0.0051	0.0051
18	3.690	4.120	3	1.6	1	1	0.07	0.053	1.286	10.31	0.0052	0.0051
19	11.070	5.240	1	1.6	1	1	0.024	0.028	0.534	11.79	0.0046	0.0051
20	11.070	5.240	2	1.6	1	1	0.032	0.028	0.534	10.52	0.0051	0.0051
21	11.070	5.240	3	1.6	1	1	0.028	0.028	0.534	9.68	0.0056	0.0051

Table S3: Results from experimental flow of 0.06 mmol PVP/0.007 mmol PEI-coated iron oxide nanoparticles (hydrodynamic size ~ 69 nm, PDI: 0.24, zeta potential: 18 mV) through hydrogel channels.

No	Nanoparticle concentration (mg/mL Fe)	Mass of nanoparticles (g)	Trial No.	Pressure at inlet (atm)	Inlet volume (mL)	Pressure at outlet (atm)	Mass loss due to adhesion (g)	Average mass loss (g)	% mass loss (Avg)	Time elapsed (s)	Velocity (m/s)	Average velocity (m/s)
1	0.011	2.008	1	1.6	1	1	0.081	0.047	2.340	10.25	0.0055	0.0059
2	0.011	2.008	2	1.6	1	1	0.052	0.047	2.34	9.09	0.0062	0.0059
3	0.011	2.008	3	1.6	1	1	0.008	0.047	2.34	9.29	0.0060	0.0059
4	0.056	2.042	1	1.6	1	1	0.059	0.044	2.154	10.26	0.0055	0.0060
5	0.056	2.042	2	1.6	1	1	0.034	0.044	2.154	8.33	0.0067	0.006
6	0.056	2.042	3	1.6	1	1	0.039	0.044	2.154	9.57	0.0059	0.006
7	0.284	2.212	1	1.6	1	1	0.013	0.024	1.085	9.89	0.0057	0.0064
8	0.284	2.212	2	1.6	1	1	0.025	0.024	1.085	8.77	0.0064	0.0064
9	0.284	2.212	3	1.6	1	1	0.034	0.024	1.085	7.86	0.0071	0.0064
10	0.583	2.424	1	1.6	1	1	0.062	0.059	2.448	9.75	0.0057	0.0055
11	0.583	2.424	2	1.6	1	1	0.041	0.059	2.448	11.31	0.0050	0.0055
12	0.583	2.424	3	1.6	1	1	0.075	0.059	2.448	9.75	0.0057	0.0055
13	1.581	3.060	1	1.6	1	1	0.116	0.067	2.179	9.23	0.0061	0.0059
14	1.581	3.060	2	1.6	1	1	0.037	0.067	2.179	9.49	0.0059	0.0059
15	1.581	3.060	3	1.6	1	1	0.047	0.067	2.179	9.63	0.0058	0.0059
16	3.690	4.120	1	1.6	1	1	0.066	0.046	1.117	9.5	0.0059	0.0060
17	3.690	4.120	2	1.6	1	1	0.026	0.046	1.117	9.3	0.0060	0.006
18	3.690	4.120	3	1.6	1	1	0.046	0.046	1.117	9.29	0.0060	0.006
19	11.070	5.240	1	1.6	1	1	0.047	0.057	1.094	9.82	0.0057	0.0063
20	11.070	5.240	2	1.6	1	1	0.038	0.057	1.094	9.41	0.0060	0.0063
21	11.070	5.240	3	1.6	1	1	0.087	0.057	1.094	7.86	0.0071	0.0063

Table S4: Results from experimental flow of 0.05 mmol PVP/0.008 mmol PEI iron oxide nanoparticles (hydrodynamic size ~ 140 nm, PDI: 0.12, zeta potential: 10 mV) through hydrogel channels.

No	Nanoparticle concentration (mg/mL Fe)	Mass of nanoparticles (g)	Trial No.	Pressure at inlet (atm)	Inlet volume (mL)	Pressure at outlet (atm)	Mass loss due to adhesion (g)	Average mass loss (g)	% mass loss (Avg)	Time elapsed (s)	Velocity (m/s)	Average velocity (m/s)
1	0.011	2.008	1	1.6	1	1	0.164	0.078	3.867	8.47	0.0055	0.0054
2	0.011	2.008	2	1.6	1	1	0.027	0.078	3.867	8.99	0.0052	0.0054
3	0.011	2.008	3	1.6	1	1	0.042	0.078	3.867	8.81	0.0053	0.0054
4	0.056	2.042	1	1.6	1	1	0.094	0.062	3.019	8.95	0.0053	0.0051
5	0.056	2.042	2	1.6	1	1	0.028	0.062	3.019	9.68	0.0049	0.0051
6	0.056	2.042	3	1.6	1	1	0.063	0.062	3.019	8.87	0.0053	0.0051
7	0.284	2.212	1	1.6	1	1	0.077	0.057	2.562	9.21	0.0051	0.0048
8	0.284	2.212	2	1.6	1	1	0.039	0.057	2.562	10.78	0.0044	0.0048
9	0.284	2.212	3	1.6	1	1	0.054	0.057	2.562	9.35	0.0050	0.0048
10	0.583	2.424	1	1.6	1	1	0.031	0.027	1.100	9.39	0.0050	0.0050
11	0.583	2.424	2	1.6	1	1	0.021	0.027	1.1	9.19	0.0051	0.005
12	0.583	2.424	3	1.6	1	1	0.028	0.027	1.1	9.65	0.0049	0.005
13	1.581	3.060	1	1.6	1	1	0.053	0.045	1.460	7.02	0.0067	0.0060
14	1.581	3.060	2	1.6	1	1	0.039	0.045	1.46	8.79	0.0053	0.006
15	1.581	3.060	3	1.6	1	1	0.042	0.045	1.46	7.95	0.0059	0.006
16	3.690	4.120	1	1.6	1	1	0.051	0.047	1.133	9.61	0.0049	0.0050
17	3.690	4.120	2	1.6	1	1	0.059	0.047	1.133	8.46	0.0056	0.005
18	3.690	4.120	3	1.6	1	1	0.03	0.047	1.133	10.39	0.0045	0.005
19	11.070	5.240	1	1.6	1	1	0.03	0.032	0.604	9.34	0.0050	0.0053
20	11.070	5.240	2	1.6	1	1	0.045	0.032	0.604	7.49	0.0063	0.0053
21	11.070	5.240	3	1.6	1	1	0.02	0.032	0.604	10.25	0.0046	0.0053

Tables S5 and S6 give a brief summary of the parameters used in our computational fluid dynamics (CFD) calculations for assessing flow and mass loss of iron oxide NPs.

Table S5: Table summarizing some key values used for CFD analysis of the lowest concentration of nanoparticles (2.008 g·mL^{-1} Fe)

Item	Specification
Type of NPs	0.07 mmol PVP and 0.005 mmol PEI-coated iron oxide NPs
Hydrodynamic diameter of NPs	144 nm
Inlet velocity	0 m/s
Average outlet velocity	0.0054 m/s
Length of tube	0.054 m
Diameter of hydrogel tube, r<br>Straight tube	0.0040 m
Reynolds number based on diameter of the tube	127.17
Density of solution	5.24 g/cc
Mass of solution at inlet	2.008 g
% Mass loss, experimental value	5.96%

<u>Method used to find Reynolds number, Re</u>

$$Re = \frac{D v \rho}{\mu}$$

where, diameter of tube (*D*) = 0.0040 m; velocity of the iron oxide NP at the outlet, *v* = 0.0054 m/s, density of the NPs, ρ = 5.24 g/cm^3 = 5.24 x 10^3 kg/m^3, and viscosity (μ) = 8.90 × 10^{-4} Pa·s

Re = 127.17

Table S6: Table summarizing some key values used for CFD analysis of the flow of iron oxide nanoparticles of concentration 4.12 g·mL^{-1} Fe.

Item	Specification
Type of NPs	0.07 mmol PVP and 0.005 mmol PEI-coated iron oxide NPs
Hydrodynamic diameter of NPs	144 nm
Inlet velocity*	0 m/s
Average outlet velocity	0.0051 m/s
Length of tube	0.054 m
Diameter of hydrogel tube, r<br>Straight tube	0.0040 m
Reynolds number based on diameter of the tube*	120.1
Density of solution	5.24 g/cc
Mass of solution at inlet	4.12 g
% Mass loss, experimental value	1.286%

<u>Method used to find Reynolds number, Re</u>

Re = $\frac{Dv\rho}{\mu}$

where, diameter of tube (D) = 0.0040 m; velocity of the iron oxide NP at the outlet, v = 0.0051 m/s, density of the NPs, ρ = 5.24 g/cc = 5.24 x 10^3 kg/m^3, and viscosity (μ) = 8.90×10^{-4} Pa·s

Re = 120.1

Figure S4 shows the size of the 69 nm sized iron oxide NPs coated with 0.06 mmol PVP/0.007 mmol PEI after being left undisturbed for six months.

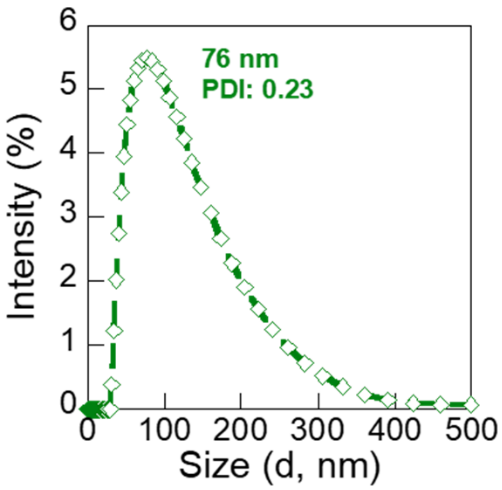

Figure S4: DLS size plot of 0.06 mmol PVP/0.007 mmol PEI-coated iron oxide NP sample solution (originally 69 nm in size) left undisturbed for six months.
